# Whole Blood Transcriptional Fingerprints of High-Grade Glioma and Longitudinal Tumor Evolution under Carbon Ion Radiotherapy

**DOI:** 10.3390/cancers14030684

**Published:** 2022-01-28

**Authors:** Maximilian Knoll, Maria Waltenberger, Jennifer Furkel, Ute Wirkner, Aoife Ward Gahlawat, Ivana Dokic, Christian Schwager, Sebastian Adeberg, Stefan Rieken, Tobias Kessler, Felix Sahm, Laila König, Christel Herold-Mende, Stephanie E. Combs, Jürgen Debus, Amir Abdollahi

**Affiliations:** 1 Department of Radiation Oncology, University Hospital of Heidelberg, Im Neuenheimer Feld 460, 69120 Heidelberg, Germany; maria.waltenberger@tum.de (M.W.); j.furkel@dkfz.de (J.F.); u.wirkner@dkfz.de (U.W.); aoife.gahlawat@dkfz-heidelberg.de (A.W.G.); i.dokic@dkfz.de (I.D.); c.schwager@dkfz.de (C.S.); sebastian.adeberg@med.uni-heidelberg.de (S.A.); stefan.rieken@med.uni-goettingen.de (S.R.); laila.koenig@med.uni-heidelberg.de (L.K.); juergen.debus@med.uni-heidelberg.de (J.D.); a.amir@dkfz.de (A.A.); 2 Clinical Cooperation Unit Translational Radiation Oncology, German Cancer Research Center (DKFZ), 69120 Heidelberg, Germany; 3 Heidelberg Institute for Radiation Oncology (HIRO), University Hospital of Heidelberg, 69120 Heidelberg, Germany; 4 German Cancer Consortium (DKTK), National Center for Tumor Diseases (NCT), German Cancer Research Center (DKFZ), 69120 Heidelberg, Germany; christel.herold-Mende@med.uni-heidelberg.de; 5 Clinical Cooperation Unit Radiation Oncology, German Cancer Research Center (DKFZ), 69120 Heidelberg, Germany; 6 Department of Neurology, University of Heidelberg Medical School, 69120 Heidelberg, Germany; tobias.kessler@med.uni-heidelberg.de; 7 Department of Neuropathology, Institute of Pathology, University of Heidelberg, 69120 Heidelberg, Germany; felix.sahm@med.uni-heidelberg.de; 8 Division of Experimental Neurosurgery, Department of Neurosurgery, University of Heidelberg Medical School, 69120 Heidelberg, Germany; 9 Department of Radiation Oncology, Technical University of Munich (TUM), Klinikum rechts der Isar, 80333 Munich, Germany; stephanie.combs@tum.de

**Keywords:** carbon ion irradiation, recurrent high-grade glioma, biomarker, liquid biopsy, whole blood transcriptome

## Abstract

**Simple Summary:**

Particle therapy with carbon ions is a promising novel option for the treatment of recurrent high-grade glioma (rHGG). Lack of initial and sequential biopsies limits the investigation of rHGG evolution under therapy. We hypothesized that peripheral blood transcriptome derived from liquid biopsies (lbx) as a minimal invasive method may provide a useful decision support for identification of glioma grade and provide novel means for longitudinal molecular monitoring of tumor evolution under carbon ion irradiation (CIR). We demonstrate feasibility and report patient, tumor and treatment fingerprints in whole blood transcriptomes of rHGG patients with pre-CIR and three post-CIR time points.

**Abstract:**

Purpose: To assess the value of whole blood transcriptome data from liquid biopsy (lbx) in recurrent high-grade glioma (rHGG) patients for longitudinal molecular monitoring of tumor evolution under carbon ion irradiation (CIR). Methods: Whole blood transcriptome (WBT) analysis (Illumina HumanHT-12 Expression BeadChips) was performed in 14 patients with rHGG pre re-irradiation (reRT) with CIR and 3, 6 and 9 weeks post-CIR (reRT grade III:5, 36%, IV:9, 64%). Patients were irradiated with 30, 33, 36 GyRBE (*n* = 5, 6, 3) in 3GyRBE per fraction. Results: WTB analysis showed stable correlation with treatment characteristics and patients tumor grade, indicating a preserved tumor origin specific as well as dynamic transcriptional fingerprints of peripheral blood cells. Initial histopathologic tumor grade was indirectly associated with TMEM173 (STING), DNA-repair (ATM, POLD4) and hypoxia related genes. DNA-repair, chromatin remodeling (LIG1, SMARCD1) and immune response (FLT3LG) pathways were affected post-CIR. Longitudinal WTB fingerprints identified two distinct trajectories of rHGG evolution, characterized by differential and prognostic CRISPLD2 expression pre-CIR. Conclusions: Lbx based WTB analysis holds the potential for molecular stratification of rHGG patients and therapy monitoring. We demonstrate the feasibility of the peripheral blood transcriptome as a sentinel organ for identification of patient, tumor characteristics and CIR specific fingerprints in rHGG.

## 1. Introduction

Carbon ion particle irradiation (CIR) is a promising novel therapy option for recurrent high grade glioma (rHGG) [1]. rHGG includes grade III and IV tumors, composed of a variety of distinct molecular subtypes [2,3] almost exclusively exhibiting a grim prognosis [4,5]. Data reported by Nyazi et al. from a German Cancer Consortium (DKTK) multi-center retrospective study of rHGG re-irradiated with photons highlighted the importance of initial tumor grade, performance score and age at reRT as important prognostic factors (re-irradiation risk score, RRRS) [6] empathizing the heterogeneity of rHGG.

CIR provides high precision particle radiotherapy modality with unique physical properties as normal tissue sparing steep dose-depth gradient [7], and specific radiobiological characteristics [8,9,10,11,12]. Its cell-killing efficacy is less dependent on the presence of oxygen [13]. The biological effects differ from conventional therapy and include the induction of a more immune-accessible environment [9], modulation of angiogenesis [9] and the eradication of tumor stem cells [8,9]. Tumor recurrences present predominantly at margins of the high-dose irradiated region following CIR, as opposed to in-field recurrences after conventional photon radiotherapy [14], supporting the observation of improved tumor cell killing by CIR. The first clinical data—comparing RRRS matched patients with photon and carbon therapy—showed promising results, especially for grade III glioma [1].

Indication for re-irradiation (reRT) is often based on imaging and clinical tumor presentation [15]. A preceding biopsy or resection is not performed regularly, thus a systematic molecular/histopathologic evaluation in the recurrent setting is not always possible. This lack of data at reRT time point as well as the non-feasibility of sequential biopsies during therapy limit the investigation of rHGG evolution and detailed examination of therapy effects.

Liquid biopsy, utilizing different blood components, such as circulating tumor cells (CTC), mutation/methylation studies on circulating free tumor DNA (cfDNA), deep single cell RNSseq and cytometric profiling of immune cells and other novel technologies may provide minimal-invasive means to dissect the molecular and characteristic features of otherwise hard to access tumors. To this end liquid biopsy using peripheral blood transcriptome as sentinel organ, is an appealing tool for translational research including patient stratification and therapy monitoring [16,17,18,19]. Peripheral blood transcriptome combined with deconvolution algorithms (e.g., CIBERSORT/TX for hematopoietic cells) may reflect tumor/therapy perturbations of cellular composition. Moreover, blood cells are exposed to drugs, traverse irradiated tissue and may elicit transcriptional responses to a plethora of factors (e.g., growth factors, immune modulators etc.) released either inherently or as function of a defined treatment by tumor cells. Therefore, in this study we aimed to evaluate the value of a specific liquid biopsy application—whole blood transcriptome analysis—to study the effects of CIR in recurrent high-grade glioma.

## 2. Materials and Methods

### 2.1. Study Cohort

Molecular information was extracted from pathology reports or physician letters. ReRT grade was extracted from radiology reports. Gray equivalent doses were calculated according to the Local Effect Model 1, LEM 1 [20]. As control cohort, data from the KORA F4 study [21,22] was retrieved from ArrayExpress (E-MTAB-1708).

### 2.2. Ethics Approval and Consent to Participate

All patient consented to participate in this study. The ethical approval was obtained by the IRB-Ethics Committee of the Medical Faculty of Heidelberg University (approval number S-540/2010).

### 2.3. Blood Processing

Whole blood, collected in PAXgene Blood RNA Tubes, was used for RNA extraction using the PAXgene Blood RNA Kit (Qiagen, Hilden, Germany). RNA quality was verified with a NanoDrop^TM^ 1000 spectrophotometer and on a Bioanalyzer (Agilent, Waldbronn, Germany). Illumina BeadChip arrays (HumanHT-12 Expression BeadChip) were used to quantify expression from 200 ng of RNA in the genomics Core Facility, DKFZ (Heidelberg, Germany).

### 2.4. CIBERSORT

Blood cell type fractions were estimated from whole blood expression data using the CIBERSORT method [23,24]. Detailed variability of CIBERSORT fractions over time is shown in Figure S5.

### 2.5. Statistical Analysis

Statistical analyses were conducted in R v 4.0.5 [25]. Low-dimensional representation of data were calculated with UMAP [26,27] and t-SNE (FI-tSNE) [28]. Non-parametric tests for repeated measurements were performed with nparLD [29], non-parametric interaction tests with the rankFD package [30]. Gene signature analysis was performed with the geneSignatures package [31]. Survival analyses (time from start of re-irradiation until death/last contact) were performed with Cox-PH models or parametric survival models (Weibull distribution) [32]. For comparison of prognostic value of tumor grade classification at different timepoints, both grade assignment at initial diagnosis (initial grade) and re-RT timepoint (reRT grade) were used with the aforementioned survival data.

The enrichR package [33] was used to perform KEGG pathway enrichment analyses (Human, 2019), pathway analyses were conducted with REACTOME [34]. Transcriptome data were log2 transformed and virtual pool normalized [35]. Lme4 was used for mixed effect model analyses [36]. Benjamini-Hochberg p-value adjustment was used for multiplicity correction (FDR). Hierarchical clustering was performed with ward.D2/complete linkage. Significance level alpha (two-sided) was fixed at 0.05 if not stated otherwise. Feature selection was performed with the modelBuildR R package [37]. Protein interaction analyses were conducted with SUMO [35]. Due to the low number of subjects included in the study, also trends are reported and/or prefiltered data (e.g., most variant genes) was analyzed. Outliers are shown in boxplots as solid dots and are identified as values below Q1 − 1.5 × IQR and above Q3 + 1.5 × IQR.

Expression data was batch normalized with ComBat [38] and z-transformed for comparative analyses (CIR + KORA F4), only genes present in both datasets were used (CIR: *n* = 34,694 genes, on both arrays: *n* = 23,437 genes). Keras [39] was used for deep learning model training.

## 3. Results

### 3.1. Study Cohort

**Figure 1:**
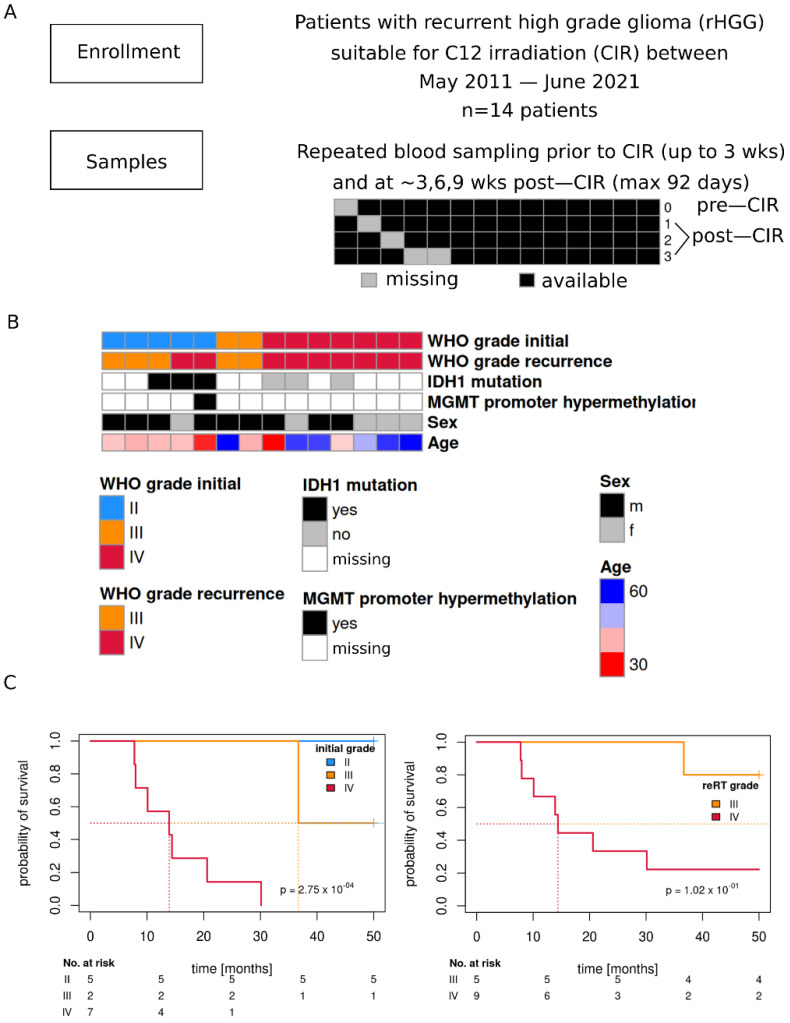
Overview of the study cohort. (**A**) Patient cohort and available data. (**B**) Distribution of main patient and molecular tumor characteristics (WHO grade initial: histopathologic grade at initial diagnosis). (**C**) Kaplan-Meier survival curves of patients (time from reRT time point to death/last follow up) for initial WHO tumor grade (left) and tumor grade at reRT time point (radiographic grade). Likelihood ratio test *p*-values, Cox-PH models.

Fourteen patients with rHGG were enrolled in the study between May 2011 and June 2021 (Figure 1A and Table 1). Blood from four time points was available for 9 of the patients, while one time point was missing for the remaining (Figure 1A). For three out of five tumors initially classified as grade II, IDH1 mutation was detected, however, for the majority of patients, MGMT promoter methylation and IDH1 mutation status was not available (Figure 1B). Initial grade II and III tumor patients were mostly male (except one), initial grade IV was more balanced (3 males, 4 females). Patients with lower grade tumors were on average younger. Initial (histopathologic) grade yielded better prognostic separation following CIR (Figure 1C).

### 3.2. Patient Characteristics Sex and Age Are Mirrored in Transcriptome

**Figure 2:**
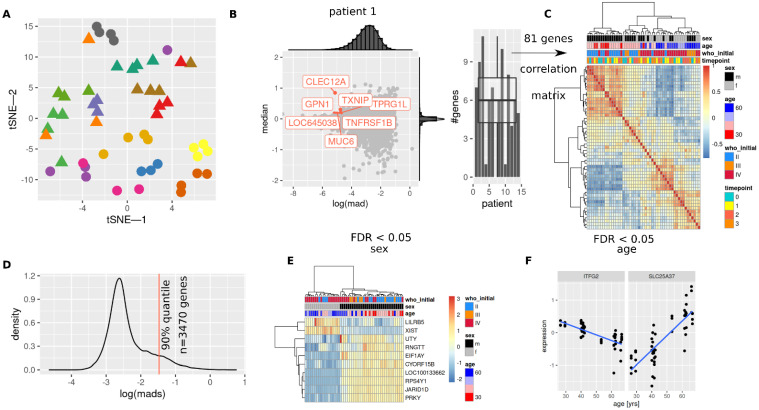
Patient characteristics reflected in whole blood transcriptome data. (**A**) t-SNE representation of gene expression data, multiple longitudinal samples per patients are assigned the same color. Triangles and dots are used solely for better discernibility. (**B**) Selection of least variant (median absolute deviation, mad) and highly expressed (median) genes per patient, left: representative distributions (mad, median) and genes for patient 1 (median: >97.5% quantile, mad: <2.5% quantile), right: numbers of selected genes for each patient. (**C**) Clustered correlation matrix of all genes identified in (**B**). (**D**) Distribution of mad values of all genes. (**E**) Genes associated with sex, (**F**) genes associated with age ((**E**,**F**) only 10% most variant genes as shown in (**D**) were evaluated, linear mixed model analysis, FDR < 0.05).

For an overview of the whole blood transcriptome, a t-SNE (Figure 2A) were computed. Repeated measurements for single patients showed mostly similar values in the respective representations (t-SNE, UMAP, Figure S1A).

To identify patient/tumor specific and thus stable features over time, least variant (mad < 2.5% quantile) and highly abundant (median > 97.5% quantile) genes were identified in serial samples per patient. Representative distributions of expression (median) and variation (mad) for a single patient is shown in Figure 2B (left), selected genes meeting the filtering criteria are labeled. Median number of analogously obtained genes were 6 per patient (0–11), and 81 unique genes in total (Figure S1B), genes per patient are shown in Table S1. Correlation and hierarchical cluster analysis of these (Figure 2C) identified similar groups of patients (clusters) associated with sex, age and initial tumor grade. Using only the most variant 10% of genes (Figure 2D), 10 genes were identified as being associated with sex (Figure 2E) and two genes with age (Figure 2F) for FDR < 0.05. The lncRNA XIST showed high expression levels in females, RPS4Y1 was among the highly expressed genes in males. XIST and JARID1D expression showed expected fractions of males/females in the KORA F4 cohort (Figure S7A). Increasing age was associated with decreasing expression levels of ITFG2 and increasing levels of SL25A37. Inverse correlation between ITFG2 and SCL25A37 was confirmed in KORA F4 data (Figure S7B).

### 3.3. Initial and Re-RT Glioma Grade Are Associated with Transcriptome Profiles

**Figure 3:**
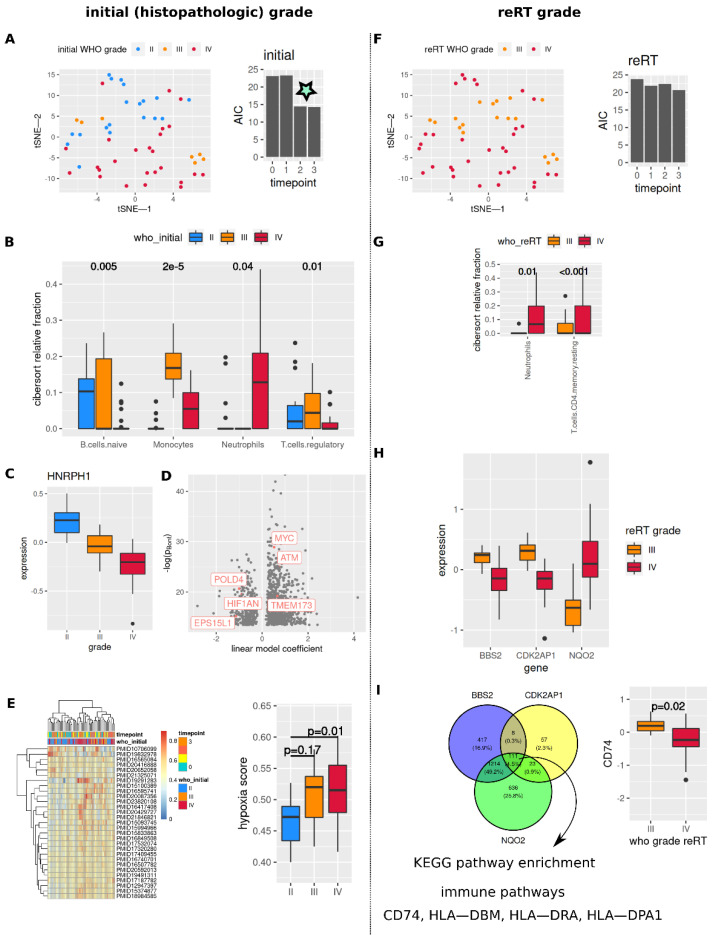
Tumor characteristics in whole blood transcriptome data. (**A**,**F**) t-SNE representation of whole blood transcriptome data (**left**) and AIC values for logistic regression models to separate grade IV vs. non-IV samples per time point ((**A**–**E**) initial WHO grade, (**F**–**I**) WHO grade at reRT time point). Star: drop in AIC for initial grades at later time points. (**B**,**G**) Differential CIBERSORT derived cell fractions (linear mixed model Wald type *p*-value, all cell fractions with *p* < 0.05). (**C**,**H**) Genes associated with tumor grade (10% most variant genes [mad], FDR < 0.05). (**D**,**I**) Genes associated with HNRPH1 (**C**) and BBS2, CDK2AP1, NQO2 (**H**) expression (Bonferroni adjusted *p*-value < 0.05, linear models). (**I**) shows a Venn diagram of commonly regulated genes from (**H**) (**left**) and CD74 expression (**right**). (**E**) Hierarchical cluster analysis of multiple hypoxia scores and association with initial grade (**right**, linear mixed model). (**B**,**C**,**G**–**I**) Dots in boxplot subfigures represent outliers (see methods).

As correlation analyses of least variable and highly expressed genes per patient (*n* = 81, Figure 2B,C) revealed similarity between samples w.r.t their initial (histopathologic) tumor grade, more detailed analyses were performed to test for associations with initial and reRT tumor grade (Figure 3). Logistic regression analyses to differentiate grade IV vs. lower grade tumors on tSNE representations (main effects: two dimensional tSNE vectors + interaction term) yielded lowest Akaike information criterion values (AIC, best separation) for late time points and initial tumor grade (Figure 3A, star, and Figure 3F).

CIBERSORT inferred cell fractions showed the lowest fractions of monocytes in initial grade II tumor samples, while naïve B cells were sparse in initial grade IV samples (Figure 3B, *p*-value < 0.05). Neutrophil fractions were higher in grade IV tumors (both initial and reRT grade, Figure 3B,G, *p*-value < 0.05). Full data is shown in Figure S2.

On the single gene expression level, HNRPH1 expression decreased in higher grade tumors (initial histopathologic tumor grade; 10% most variable genes, FDR < 0.05, linear model, Figure 3C). *n* = 1141 genes showed significant association with HNRPH1 expression (Bonferroni adjusted *p*-value < 0.05, Figure 3D). Form these, 894 (78%) were present in the KORA F4 dataset, 746 (83%) showing significant association with HNRPH1 expression (Figure S7C). Among these were genes involved in DNA damage repair (POLD4, ATM), hypoxia sensing (HIF1AN), and inflammatory signaling (STING/TMEM173) (Figure 3D). KEGG pathway analysis of common associated gene sets (CIR + KORA F4) identified DNA replication, T-cell receptor, NFkB and HIF-1 signaling (Figure S7C, right). Association with hypoxia was further assessed by calculating hypoxia scores from a set of hypoxia signatures (Figure 3E, left, each row corresponds to a score based on a published hypoxia signature), showing higher median hypoxic scores in higher grade tumors (Figure 3E, right). Associations of HNRPH1 with covariates for the KORA F4 dataset are shown in Figure S7D, observed absolute correlations were mostly <0.2.

ReRT grade of tumors showed association with three genes (10% most variant genes, FDR < 0.05, Figure 3H). Analogously to Figure 3D, associations of these three candidates with expression of all genes was assessed (Bonferroni adjusted *p*-value < 0.05) yielding 111 consensus genes (Figure 3I). KEGG pathway enrichment analysis of these 111 candidates highlighted MHC-II genes HLA-DBM, HLA-DRA, HLA-DPA1 in identified pathways (Figure S3), CD74 (the HLA class II histocompatibility antigen gamma chain) was lower expressed in reRT grade IV tumors (Figure 3I, right). Associations in KORA F4 data are shown in Figure S7E.

Finally, we aimed to confirm the presence of a disease signature in the CIR cohort samples by comparing transcriptome profiles to data from the KORA F4 study (Figure S7F). A 2-dimensional umap representation did not reveal a clear separation of both cohorts (Figure S7, left). A deep learning model however, was able to identify samples belonging to the CIR cohort with an AUC of 77% (95% CI: 65–89%, Figure S7F, right).

### 3.4. CIR Induces Sustained and Transient Changes in Transcriptome

**Figure 4:**
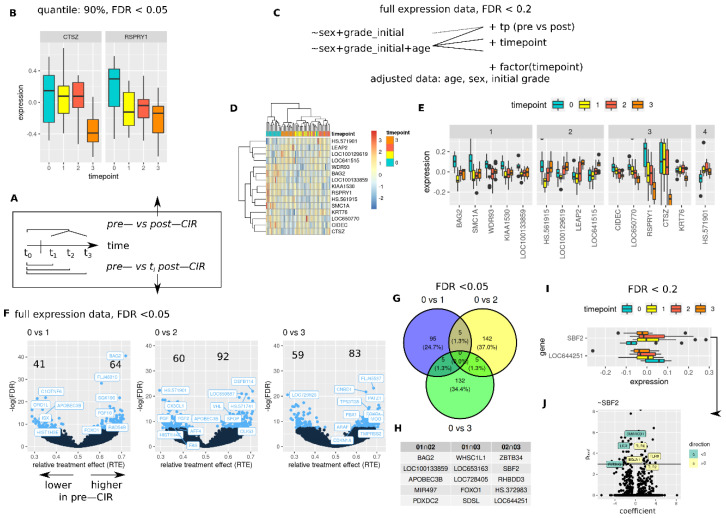
CIR induced transcriptome alterations, (**A**) gives an overview of outlined analyses. (**B**) Differential genes in pre- vs. post-CIR, ~factor(timepoint), linear mixed model, most variant 10% genes (mad). (**C**) Models tested with more liberal cutoffs (FDR < 0.2, all genes). Hierarchical cluster analysis (**D**) of all genes identified in (**C**) on age, sex and initial WHO grade adjusted expression data, (**E**) genes grouped by expression dynamics (see text, (**D**,**E**) z-transformed adjusted data). (**F**) Pairwise differences between pre and nth post-CIR time point, non-parametric model analysis (nparLD, FDR < 0.05). (**G**) Venn diagram and table of genes (**H**) from (**F**) with FDR < 0.05. (**I**) Commonly identified genes with FDR < 0.2 between all pairwise comparisons (see **F**), and genes associated with SBF2 expression ((**J**), Bonferroni adjusted *p*-value < 0.05) (no associated genes were detected for LOC644251).

**Figure 5:**
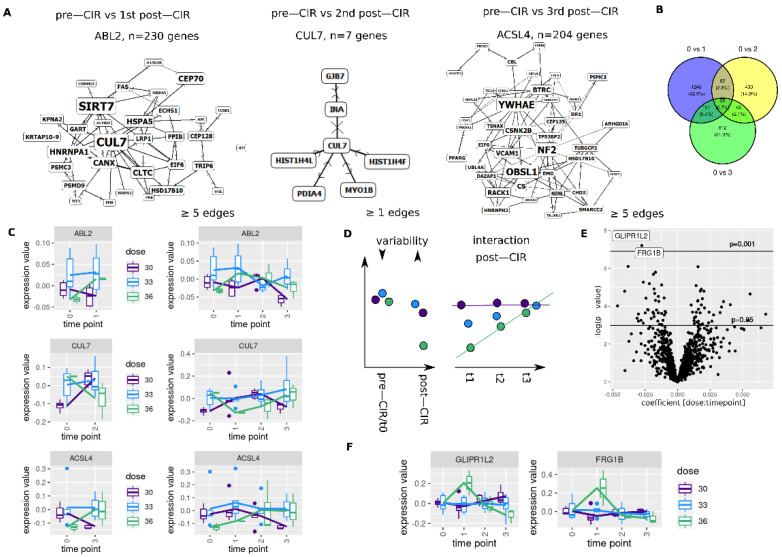
Carbon irradiation dose dependent associations in whole blood transcriptome. (**A**) Protein interaction networks of genes with significant interaction between pre-CIR and nth post-CIR time point (rankFD, FDR < 0.05). (**B**) Venn diagram of identified genes. (**C**) Gene expression of identified network proteins. (**D**) Schematics of tests for least variant pre-CIR genes (**left**) with dose dependent interaction (**right**). Arrows indicate degree of variability. (**E**) Interaction effects for genes with low variation pre-CIR (pairwise TOST between dose levels) and linear mixed model analyses in post-CIR samples. Candidates with lowest *p*-values are shown in (**F**).

CIR effects were evaluated independently from applied dose (Figure 4), and for dose dependent changes (Figure 5). Dose independent tests were performed as global tests (any difference between pre- and post-CIR samples), and as pairwise tests (pre-CIR vs. nth post-CIR time point, Figure 4A). Evaluation of most variant 10% of genes identified CTSZ and RSPRY1 as late (last time point) and early (first time point post-CIR, no return to baseline) changing genes (Figure 4B, FDR < 0.05).

More liberal analysis (FDR < 0.2 on full expression data) with adjustment for sex, age, and initial tumor grade (Figure 4C) identified groups of genes with distinct dynamics post-CIR (Figure 4E) and similar expression patterns within the respective time points (Figure 4D). One group showed a decrease and sustained lower expression post-CIR (Figure 4E, (1), a decrease followed by return to baseline or even surpassing initial expression levels (2), steady or late decrease (3) and early sustained increase (4). Note that the data in Figure 4D and E is age, sex and initial grade adjusted, in contrast to data shown in Figure 4B.

Pairwise differences (control vs. nth time point) calculated from full expression data are shown in Figure 4F (non-parametric analysis, full expression data, FDR < 0.05). Top candidates are labeled based on lowest FDR, occurrence in COSMIC cancer mutation census candidates (7 May 2021) or association with DNA repair.

No genes were found as differentially regulated between the three groups for a FDR < 0.05 (Figure 4G), however, for FDR < 0.2, two genes (SBF2 and LOC644251) were identified (Figure 4I). Overlapping genes between two pairwise comparisons are shown in Figure 4G,H.

SBF2 showed a sustained increase in expression post-CIR. Positively associated genes included toll like receptors, whereas negative associations were observed for DNA repair gene LIG1, SMARCD1 and FLT3LG (all genes, linear model analysis, Bonferroni *p*-value < 0.05, Figure 4J). Expression of labeled genes for non-adjusted and adjusted data (sex, age, initial tumor grade) is shown in Figure S4.

### 3.5. CIR Leads to Dose Dependent Transcriptome Alterations

Next, we assessed if CIR dose has an impact on the transcriptome (Figure 5). Testing for an interaction between dose and two time point (pre-CIR and *n*th post-CIR) yielded *n* = 1507 (0 vs. 1), *n* = 595 (0 vs. 2) and *n* = 1149 (0 vs. 3) genes with an FDR < 0.05 (rankFD, Wald-type statistic, interaction *p*-value). The corresponding Venn diagram is shown in Figure 5B. Resulting gene sets were tested for protein interaction networks (SUMO), largest identified networks are shown in Figure 5A (networks: ABL2: 0 vs. 1, CUL7: 0 vs. 2, ACSL4: 0 vs. 3).

Expression values for the identified network genes are shown in Figure 5C. For high dose, ABL2 shows expression increase with return to baseline, CUL7 shows a transient decrease. ACSL4 shows a delayed increase.

To more systematically capture different dynamics of gene expression (see Figure 4E), the post-CIR part was also analyzed separately from the pre-CIR data (Figure 5D), while restricting the evaluated gene set to candidates with equivalent expression in pre-CIR time point (*n* = 1024, pairwise equivalence tests between dose levels, TOST, *p* < 0.05).

Interaction terms for these genes are shown in Figure 5E, with expression levels of candidates with *p*-values < 0.001 (non-adjusted, linear mixed model analysis without pre-CIR data) shown in Figure 5F. Both genes—GLIPR1L2 and FRG1B—show a steep, transient increase post-CIR for high dose. All candidates with *p* < 0.05 are shown in Table S2.

### 3.6. Longitudinal Transcriptome Analysis Identifies Two Trajectories

**Figure 6:**
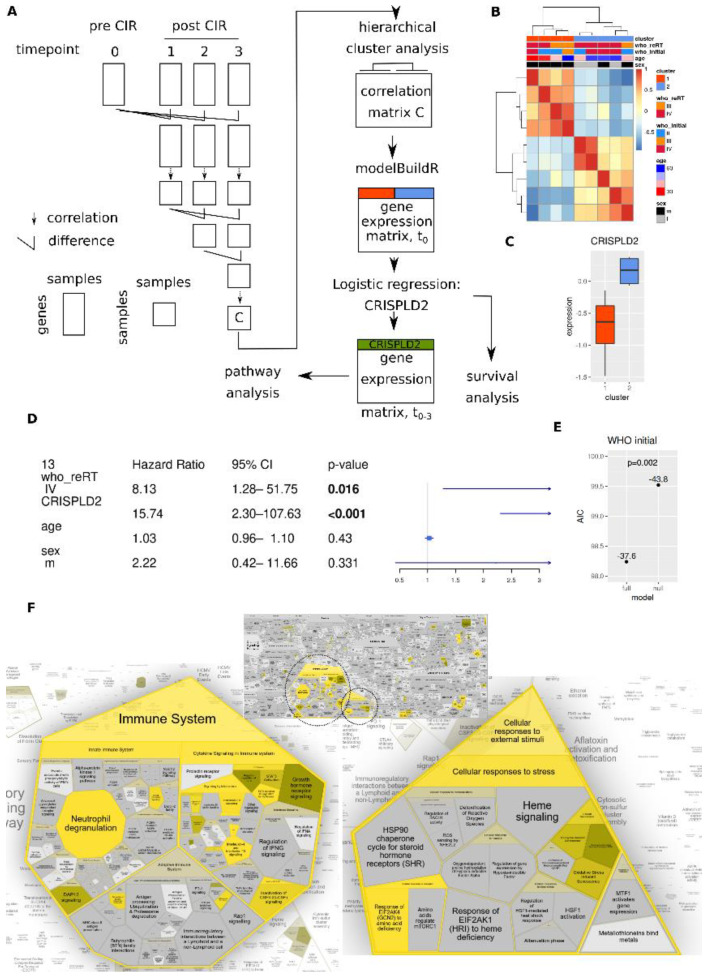
Assessment of longitudinal changes (dynamic profiles) in whole blood transcriptomes. (**A**) Schema of performed calculations. (**B**) Hierarchical cluster analysis of the correlation matrix C. (**C**) CRISPLD2 expression in main clusters shown in C. (**D**) Multivariate survival analysis (parametric survival regression, Weibull distribution) with tumor grade at reRT time point. (**E**) AIC of multivariate survival models (analogously to **E**) with initial tumor grade instead of reRT grade, null model: without CRISPLD2, full model: with CRISPLD2. (**F**) REACTOME pathway analysis of genes associated with CRISPLD2 expression (Bonferroni adjusted *p*-value < 0.05, *n* = 834 genes).

Overall changes in gene expression profiles was assessed by repeated evaluation of differences and correlations (Figure 6A). This approach identified two main groups of patients (Figure 6B) that could be separated by CRISPLD2 expression at the pre-CIR time point (Figure 6C). Further differential genes are shown in Figure S6.

Multivariate survival analysis with age, sex and reRT WHO grade revealed that CRISPLD2 expression was an independent prognostic marker (Figure 6D). It also improved the model performance when substituting reRT with initial WHO grade (Figure 6E).

Finally, REACTOME analysis of genes associated with CRISPLD2 expression (*n* = 834, Bonferroni *p*-value < 0.05) identified two main areas: immune system and cellular response to external stimuli (Figure 6F). The former included STAT5 activation, FLT3, IL4 and 13 signaling, the latter genes associated with senescence phenotype. KEGG pathway analysis also highlights a number of immune-associations (Table S3).

## 4. Discussion

Treatment of recurrent high-grade glioma remains a therapeutic challenge [4,5]. Conventional photon re-irradiation might be limited due to a substantial risk of normal-tissue toxicities and radio necrosis [40]. Here, particle therapy with carbon ions (CIR) confers an alternative due to its physical [7] and biological properties [8].

In this study, we evaluated whole blood transcriptomics as minimal invasive method to study the effects of CIR in recurrent high-grade glioma. To our knowledge, this is the first study incorporating longitudinal analysis of high-grade gliomas treated with CIR.

As blood cells also patrol the tumor, they can exert local (e.g., cytotoxic T cells) and distant effects via cytokines and growth factors on the bone marrow and hematopoietic homeostasis. One might hypothesize that such effects might be conserved and mirrored in the easily accessible sentinel organ blood (respective gene expression profiles of circulating cells and/or circulating RNAs) [41,42,43,44,45,46]. Importantly, we assume that the observed changes following therapy are predominantly indirect effects mostly on leucocytes, arising from tumor and irradiation interaction, and only to a lesser degree (if at all) a direct measurement of changes in circulating tumor cells.

Effects especially on circulating T-cells following irradiation have long been known [47,48,49,50]. However, our analyses (whole blood transcriptome) were not confined to specific subpopulations of cell types but rather constitute a mixture of all present cells. We evaluated associations with patient, tumor and treatment characteristics on transcriptome level and used CIBERSORT [23,24] to estimate cell fractions from bulk expression data.

Individualized transcriptome fingerprints, however, can be assumed to be a composition of healthy inter-subject variation (human genetics) and disease (tumor and/or other disease) specific alterations. We therefore tried to separate these effects, starting by testing for non-tumor patient characteristics like age and sex: higher XIST expression was observed in females [51,52], and higher RPS4Y1 expression in males. These two genes have been reported to be linked to gender in single cell analyses [53]. ITFG2, which was observed to be less expressed with increasing age, is part of the KICSTOR complex which has been linked to aging and which modulates mTOR [54,55].

Next, we assessed the disease specific transcriptome fingerprint within the highly heterogeneous group of rHGG (grade III, IV, each comprising a variety of distinct molecular subtypes). Interestingly, we found transcriptome profiles linked to initial (histopathologic) determined tumor grade (grade II to IV) by evaluation of least variant genes per patient. Together with the observed improved prognostic separation of patients based on the initial (histopathologic) grade as compared to clinical/radiographically determined grade at reRT time point, this hints to a persistent transcriptome fingerprint of the initial tumor, preserved even years after initial diagnosis (up to 17.7 years). Furthermore, transcriptome profiles could be better separated with initial (histopathologic) grade IV tumors vs. non-grade IV as compared to reRT (radiographic/clinical) tumor classification after a latency period following CIR (second and third time point post-CIR). This hints towards a differential response to CIR depending on initial tumor grade (see also below).

An increased expression of HNRPH1 correlated with initial (histopathologic) tumor grade. HNRPH1 has been described in colon cancer development [56,57], and in the splicing oncogenic switch [58]. We found expression of HNRPH1 to be associated with genes involved in DNA repair (ATM, POLD3), hypoxia (HIF1AN) and inflammatory signaling (TMEM173/STING), with the latter showing higher expression levels in lower initial grade tumors. Higher activity of STING mediated IFN-I signaling has been reported to enhance anti-glioma immunity [59], a direct link between tumor grade and STING activity, however, has not been established yet. Higher hypoxia signature scores were observed in initial grade IV tumors, which might mirror the highly hypoxic state within glioblastoma [60,61].

Clinical/radiographic determined glioma grade at reRT time point was associated with differential expression of CDK2AP1 which has been implicated in glioma tumorigenesis (putative tumor suppressor) [62]. Further associations were found for MHC-II genes: CD74, e.g., was less expressed in reRT grade IV tumors. CD74 expression has been reported to be confined to microglia/macrophages in glioma [63] and as marker associated with response to TMZ therapy [64], highlighting the interdependency between immune system and therapy efficacy.

Deconvolution of immune cells using CIBERSORT revealed higher neutrophil counts in higher grade tumors as reported previously [65]. Higher inferred fractions of monocytes were only observed when evaluating differences based in initial (histopathologic) grade classifications. Monocyte abundance has been linked to levels of tumor associated macrophages (TAMs) within tumors [66]. Thus, one might hypothesize that detailed subtyping of circulating monocytes might help to gain insights into composition of TAMs with their diverging impacts on tumor development [9].

We performed comparison of samples from our CIR cohort against data from the KORA F4 study [21,22]. Importantly, only 68% of genes from the CIR dataset were present in the latter, which might be the reason for the challenging separation of samples from both cohorts. Nevertheless, using a deep learning approach we achieved a satisfactory AUC given the small CIR dataset.

Next, we assessed the dynamic aspects of treatment associated transcriptome changes, comprising time-dependent alterations (pre- vs. post-CIR) and differences associated with applied dose. We observed qualitatively different time-dependent effects, i.e., transient or sustained changes in gene expression at early or late time point post-CIR. This indicates a pleiotropy of CIR induced changes, potentially leading to a long-lasting CIR fingerprint.

Among the differentially expressed genes following CIR, were genes involved in the regulation of metastatic ability (BAG2 [67]), immunological modification (neopeptide generation/modification, APOBEC3B [68,69] and TLR3 dependent NK cell modulation, RHBDD1 [70]) as well as the transcription factor FOXO1 [71].

We observed lasting alternations in SBF2, which was associated with expression of toll like receptors, DNA-repair machinery, PI3K signaling (FLT3LG) and chromatin accessibility genes (SMARCD1). FLT3LG seems to exert its anti-tumor activity in conjunction with macrophages and CD4 + T-cells in glioma [72], supporting the observation of a specific immune-modulatory effect of CIR [9].

Dose dependent changes revealed transient and sustained changes in gene expression. Protein interaction network analysis revealed co-regulation of three main networks for each time point (vs. pre-CIR). For first post-CIR time point, a network centered on the ABL proto-oncogene 2, non-receptor tyrosine kinase (ABL2) was identified, with highly connected components SIRT7 and CUL7, all being implicated in tumorigenesis [73,74,75,76]. Second post-CIR time point identified cullin 7 (CUL7) and last time point acyl-coa synthetase long chain family member 4 (ACSL4) with NF2, OBSL1 and YWHAE as highly connected components, all being described or hypothesized to play roles in tumor development [77,78,79,80,81]. In addition, the tp53 target GLIPR1L2 [82] was found to be transiently regulated for high dose. In summary, these findings hint towards differential CIR dose-dependent biological effects; however, future studies are warranted which evaluate in detail the contribution of the biological tumor background (high molecular heterogeneity in our cohort) and irradiated tumor/blood volume (not considered here).

Finally, we performed a more global assessment of transcriptome changes over time. Interestingly, we observed two clearly separated groups of tumors with two distinct changes of gene expression profiles over time. Initial separation at pre-CIR time point of these two groups was feasible based on CRISPLD2 expression. Expression of the latter had a prognostic value in addition to the factors tumor grade (both initial histopathologic and reRT clinical/radiographic). CRISPLD2 expression associated genes were linked to the immune system and cellular responses to external stimuli, especially senescence, pathways. Its expression has been reported as inhibiting proinflammatory mediators in lung epithelial cells and fibroblasts [83]. REACTOME analyses further indicated IL4/IL3 pathways among associated genes, which can induce a shift towards immune-suppressive M2 phenotype of macrophages [84], linked to poor outcome in glioma [9,85]. Thus, evaluation of longitudinal transcriptome changes post-CIR might have captured two groups of tumors with differential immunological state.

Main limitations of our study are the low number of subjects and the highly heterogeneous study cohort. Different analysis techniques—e.g., FACS quantification of specific cell populations, or RNA sequencing/PCR based quantification of single genes—should be implemented to overcome shortcomings of the herein utilized methods. In addition, evaluation of alternative treatment/control groups are warranted to further decipher treatment induced vs. normal disease development caused alterations. Thus, further studies are warranted to validate our findings in independent cohorts.

## 5. Conclusions

In this study, we assessed the value of a liquid biopsy (lbx) approach using whole blood transcriptome analysis for characterization of patients, tumor biology and particle irradiation with Carbons (CIR) in recurrent high-grade glioma (rHGG) patients. We observed that stable molecular tumor characteristics such as initial tumor grade are preserved even years after the initial diagnosis, that CIR irradiation leads to transient and long-term changes in whole blood transcriptome and that the evaluation of patterns of longitudinal change might help decipher rHGG heterogeneity and interactions with the immune system. Thus, the proposed liquid biopsy approach might be a versatile tool to study tumor characteristics, tumor evolution and treatment effects.

## Figures and Tables

**Table 1:** Patient characteristics. % at reRT time point, ! at initial diagnosis time point.

Feature		N (%)/Time [Month]
All		14 (100)
Sex		
	Male	9 (64)
	Female	5 (36)
Age [yr] %		
	<40	2 (14)
	40–49	6 (43)
	≥50	6 (43)
Grade !		
	II	5 (36)
	III	2 (14)
	IV	7 (50)
Grade %		
	III	5 (36)
	IV	9 (64)
Grade ! -> %		
	II -> III	3 (21)
	II -> IV	2 (14)
Time to reRT		
	II	5 (36)/80.9
	III	2 (14)/86.8
	IV	7 (50)/11.4
C12 irradiation		
Total dose [GyRBE]/#fx	30/10	5 (36)
	33/11	6 (43)
	36/12	3 (21)
Single dose [GyRBE]	3	14 (100)

## Data Availability

Available from the authors upon reasonable request.

## Supplementary Materials

The following are available online at https://www.mdpi.com/article/10.3390/cancers14030684/s1, Figure S1: UMAP representation of transcriptome data and least variable highly expressed genes. Figure S2: CIBERSORT fractions for initial histopathologic and reRT tumor grade. Figure S3: KEGG pathway enrichment analysis of *n* = 111 consensus genes for re-RT grade association Figure S4: Selected gene expression over time for non-covariate adjusted and adjusted expression data. Figure S5: Variability of CIBERSORT estimated cell fractions. Figure S6: Volcano plot showing differential genes between both identified trajectories/clusters. Figure S7: Evaluation of reported findings in data from the KORA F4 study. Table S1: Least variant and highly abundant expressed genes in serial samples per patient. Table S2: Differentially expressed genes post-CIR with a significant interaction term between time and dose effects and equivalent expression at pre-CIR time point. Table S3: KEGG pathway enrichment for gene expression associations with CRISPLD2.
