# Supplementary material for: Whole Blood Transcriptional Fingerprints of High-Grade Glioma and Longitudinal Tumor Evolution under Carbon Ion Radiotherapy
Source: Cancers (Basel). 2022 Jan 28;14(3):684. doi: 10.3390/cancers14030684 (PMC8833402; doi:10.3390/cancers14030684)
Supplement: Supplementary file 1 [file cancers-14-00684-s001.zip › cancers-1439087-supplementary/Table S1.pdf]

Table S1: Least variant and highly abundant expressed genes in serial samples per patient.

Patient 1	Patient 2	Patient 3	Patient 4	Patient 5	Patient 6	Patient 7
CLEC12A<br>GPN1<br>LOC645038<br>MUC6<br>TNFRSF1B<br>TPRG1L	DERL1<br>EXOSC10<br>GPX7<br>HLA.A<br>HNRPA2B1<br>LOC647099<br>LOC730316<br>PALLD<br>USP9Y	AUTS2<br>CFD<br>EZH1<br>HS.195033<br>IPO8<br>LOC100133772<br>LOC283267<br>LOC400721<br>NUP93<br>OLFM1<br>WDR18	ADNP	HERC1<br>ISOC1<br>KLHDC3<br>MAT2B<br>OXSR1<br>RBM22	C9ORF142<br>CD151<br>CD5<br>ERF<br>GAB3<br>PYCARD<br>RIPK1	none
Patient 8	Patient 9	Patient 10	Patient 11	Patient 12	Patient 13	Patient 14
ATXN7L2<br>DUSP22<br>LOC729439<br>RASAL3<br>SAFB2<br>TIMM22	ANXA5<br>CYB5R4<br>DIRC2<br>F8A1<br>HAUS4<br>HS.57079<br>LOC646463<br>PIAS1<br>TYROBP<br>ZBTB34	MT2A	ANP32A<br>FKBP14<br>GPBP1L1<br>LOC100134159<br>LOC391075<br>LOC645895<br>TUBA4A<br>ZHX2	CCDC102A<br>HS.534439<br>LOC731096<br>ZNF212	FAM65B<br>FLJ22662<br>GDE1<br>LOC388458<br>LOC390671<br>STAT5B	CLDND1<br>FAM38A<br>GIMAP5<br>LOC641814<br>SLC38A1
