# Supplementary material for: Whole Blood Transcriptional Fingerprints of High-Grade Glioma and Longitudinal Tumor Evolution under Carbon Ion Radiotherapy
Source: Cancers (Basel). 2022 Jan 28;14(3):684. doi: 10.3390/cancers14030684 (PMC8833402; doi:10.3390/cancers14030684)
Supplement: Supplementary file 1 [file cancers-14-00684-s001.zip › cancers-1439087-supplementary/Table S2.pdf]

Table S2: Differentially expressed genes post CIR with a significant interaction term between time and dose effects and equivalent expression at pre-CIR time point.

estimate dose:timepoint	p-value	ID						
-0.031509346	0.00044376	GLIPR1L2	-0.014499574	0.01424727	CTU2	0.067870974	0.02795379	RAB10
-0.02753822	0.00074377	FRG1B	0.035381515	0.01431469	METTL3	0.027758173	0.02892108	WRNIP1
-0.03810066	0.00223977	C19ORF21	0.04060999	0.01695807	ACTL6A	-0.010111741	0.02923806	LOC100128574
0.015766616	0.00227792	MARCH10	0.018352105	0.0171443	PREI3	-0.018040762	0.03172337	HS.554965
-0.016915346	0.00304165	KLF15	0.010495628	0.01716938	LOC729394	0.014011017	0.03193308	SNORD4B
-0.018611723	0.00339486	KCNA7	-0.046179063	0.01791577	TMEM101	0.012957547	0.03403211	ALDH3A2
-0.026318929	0.00513187	HS.584126	0.033871843	0.01910312	CPNE8	-0.031209051	0.03447625	ZRANB1
-0.021658243	0.00556301	LOC100130413	0.020766306	0.01997855	HS.581690	-0.020322277	0.03510736	HLA-C
-0.012220573	0.00594731	LOC541469	0.016134634	0.02141459	TMEM19	-0.011258044	0.03844626	LOC100133189
0.014257361	0.00616712	CCL19	-0.023014936	0.02196883	LOC644507	0.054477159	0.03851022	PPP1CC
-0.018090974	0.00736673	LOC647716	-0.022597334	0.0221836	TRIM43	-0.028151374	0.03857183	NDST2
0.018335344	0.00747691	EPC2	-0.016198601	0.02229001	LOC652615	0.012684192	0.03937628	HS.168950
0.017456606	0.00824648	FLJ40292	-0.009654708	0.02405437	HOXA3	-0.030174984	0.04030301	ATXN7L3
0.020343821	0.00829982	CCNJ	0.011596216	0.0249194	C4ORF35	-0.015170725	0.04031267	HS.554701
-0.040760455	0.0083233	JARID1A	0.024305008	0.02511119	GXYLT1	0.026455853	0.04047476	USP36
-0.017641821	0.00931468	KIAA0895	0.014516256	0.02527473	MIR548F2	0.016119688	0.04074224	HS.550095
0.055924672	0.00962438	SDHD	-0.018884637	0.0253409	C21ORF96	0.014915998	0.04232289	HS.543130
-0.014739931	0.01094392	LOC644277	-0.009033341	0.02552176	NTN4	0.023984355	0.04387862	COQ4
-0.01036251	0.01187292	PIP	0.032749298	0.02558875	SYNCRIP	-0.026369758	0.04480775	TM7SF3
-0.024371215	0.01196697	F12	-0.014752262	0.02594448	LOC642954	0.012962009	0.0460197	HPS5
0.019101996	0.01231227	PTPN18	0.017370374	0.02598677	FXR1	0.019303229	0.0467126	STX6
-0.017087238	0.01340994	IKZF2	-0.024929958	0.02604518	HS.355933	-0.012512833	0.04789284	TRIM41
0.015746303	0.0136847	LOC729242	-0.01224447	0.02684768	C9ORF50	0.016464088	0.04808994	HYPB
0.015396619	0.01389714	LCE4A	-0.018386894	0.02691483	COG7	-0.014004009	0.04824768	C3ORF43
			-0.030146134	0.02764808	POLR2J	0.018090456	0.04904604	ZDHHC6
