# Supplementary material for: Whole Blood Transcriptional Fingerprints of High-Grade Glioma and Longitudinal Tumor Evolution under Carbon Ion Radiotherapy
Source: Cancers (Basel). 2022 Jan 28;14(3):684. doi: 10.3390/cancers14030684 (PMC8833402; doi:10.3390/cancers14030684)
Supplement: Supplementary file 1 [file cancers-14-00684-s001.zip › cancers-1439087-supplementary/Table S3.pdf]

Table S3: KEGG pathway enrichment for gene expression associations with CRISPLD2 (Bonferroni adjusted p-value < 0.05).

Term	Overlap	P.value	Adjusted.P.value
Osteoclast differentiation	26/127	1.30860845970941e-11	3.58558717960379e-09
Tuberculosis	27/179	6.89430660530722e-09	9.44520004927089e-07
T cell receptor signaling pathway	18/101	1.73667647182962e-07	1.58616451093772e-05
Autophagy	20/128	3.42500695203276e-07	2.34612976214244e-05
Fc gamma R-mediated phagocytosis	16/91	1.0072955209707e-06	5.51997945491942e-05
Leishmaniasis	13/74	1.04167067240096e-05	0.000475696273729774
Phagosome	18/152	6.52812437580468e-05	0.00233206113435819
HIF-1 signaling pathway	14/100	6.80893761856407e-05	0.00233206113435819
Fc epsilon RI signaling pathway	11/68	0.000108167033116561	0.00329308523043752
Chemokine signaling pathway	20/190	0.000138413965072347	0.00354577045574635
Th17 cell differentiation	14/107	0.000143210861757376	0.00354577045574635
NF-kappa B signaling pathway	13/95	0.000155289217039986	0.00354577045574635
Hematopoietic cell lineage	13/97	0.000192144635269415	0.0040498176972169
Ribosome	17/153	0.000225265286784113	0.00440876346991764
Parathyroid hormone synthesis, secretion and action	13/106	0.000465312437872512	0.00849970719847121
Estrogen signaling pathway	15/137	0.000603739443005331	0.0103390379614663
B cell receptor signaling pathway	10/71	0.000693781200703556	0.0111821205289867
Toxoplasmosis	13/113	0.000859964241610623	0.0119874405862161
Pathways in cancer	38/530	0.000866547665588311	0.0119874405862161
Salmonella infection	11/86	0.000874995663227451	0.0119874405862161
Natural killer cell mediated cytotoxicity	14/131	0.00115001750399534	0.0149589877923537
Chagas disease (American trypanosomiasis)	12/103	0.00120108661106489	0.0149589877923537
NOD-like receptor signaling pathway	17/178	0.00128463213672308	0.0153038784983532
Th1 and Th2 cell differentiation	11/92	0.0015344404051396	0.0175181946253437
Lysosome	13/123	0.001881627864439	0.019960446167397
Legionellosis	8/55	0.00189405693559242	0.019960446167397
Kaposi sarcoma-associated herpesvirus infection	17/186	0.00206436877407865	0.0207570141316483
TNF signaling pathway	12/110	0.00212115472878158	0.0207570141316483
Renal cell carcinoma	9/69	0.00219768669434676	0.0207643501465866
Prostate cancer	11/97	0.00235405813580237	0.0215003976403283
JAK-STAT signaling pathway	15/162	0.00325262063696455	0.0287489695009125
Human immunodeficiency virus 1 infection	18/212	0.00346210777841067	0.0296442978526413
C-type lectin receptor signaling pathway	11/104	0.00406270009833531	0.0337327220286022
Human T-cell leukemia virus 1 infection	18/219	0.00486357390680243	0.0391946838371725
Influenza A	15/171	0.00538621254051976	0.0421663496029261
Platelet activation	12/124	0.00567257579747447	0.0431746046807779
Acute myeloid leukemia	8/66	0.00598735457752562	0.044338787952487
Leukocyte transendothelial migration	11/112	0.00709358897003183	0.0503531994480703
Epithelial cell signaling in Helicobacter pylori infection	8/68	0.00716706123530927	0.0503531994480703
Adipocytokine signaling pathway	8/69	0.00781878380712017	0.0535586690787732
Hepatitis B	14/163	0.0084111364820251	0.0562110096603629
Ras signaling pathway	18/232	0.00869003326791184	0.0565386778057397

ErbB signaling pathway	9/85	0.00887285819579127	0.0565386778057397
Neurotrophin signaling pathway	11/119	0.0109872129077676	0.0671911594516675
Gap junction	9/88	0.0110350444354928	0.0671911594516675
Alzheimer disease	14/171	0.0125041188573839	0.074481055802678
Pertussis	8/76	0.0136949811977639	0.0798388265571769
Vibrio cholerae infection	6/50	0.0172173100921944	0.0982821451096097
Necroptosis	13/162	0.0182847738987845	0.102245470372795
Non-small cell lung cancer	7/66	0.0197218060499842	0.108075497153913
Relaxin signaling pathway	11/130	0.0201609451129459	0.108315665900925
Viral carcinogenesis	15/201	0.0215232523519786	0.113410983546964
FoxO signaling pathway	11/132	0.0223027768604668	0.115301148297507
Staphylococcus aureus infection	7/68	0.0228746434018664	0.115555483826447
Thyroid hormone signaling pathway	10/116	0.0231954438337759	0.115555483826447
Ferroptosis	5/40	0.0245074879898881	0.11991163766481
Prolactin signaling pathway	7/70	0.0263589998283732	0.12267994731258
Human cytomegalovirus infection	16/225	0.0268270974082945	0.12267994731258
Colorectal cancer	8/86	0.026919935179407	0.12267994731258
Bladder cancer	5/41	0.02698460719158	0.12267994731258
Endocytosis	17/244	0.0273119590732387	0.12267994731258
Measles	11/138	0.0297252703739764	0.13129602628221
Adherens junction	7/72	0.0301885023933549	0.13129602628221
Endometrial cancer	6/58	0.0332406997558686	0.142311745829812
Pentose phosphate pathway	4/30	0.0347701501590077	0.146569556054894
Insulin resistance	9/108	0.0365872034933853	0.151889115034397
Apoptosis	11/143	0.0371407690047613	0.151889115034397
Long-term depression	6/60	0.0383779765974974	0.154640670407563
Cytokine-cytokine receptor interaction	19/294	0.0398292463346476	0.155369243778633
Axon guidance	13/181	0.0401846984731531	0.155369243778633
GnRH signaling pathway	8/93	0.0402599135338793	0.155369243778633
MAPK signaling pathway	19/295	0.0409848020458839	0.155969941119058
Proteoglycans in cancer	14/201	0.0424532234879897	0.159344975831633
Cholinergic synapse	9/112	0.0445108954673587	0.164810612946707
Serotonergic synapse	9/113	0.046657706434762	0.170456154174997
Notch signaling pathway	5/48	0.0487409497440119	0.175723950392885
