# Supplementary figures and images for: Whole Blood Transcriptional Fingerprints of High-Grade Glioma and Longitudinal Tumor Evolution under Carbon Ion Radiotherapy
Source: Cancers (Basel). 2022 Jan 28;14(3):684. doi: 10.3390/cancers14030684 (PMC8833402; doi:10.3390/cancers14030684)

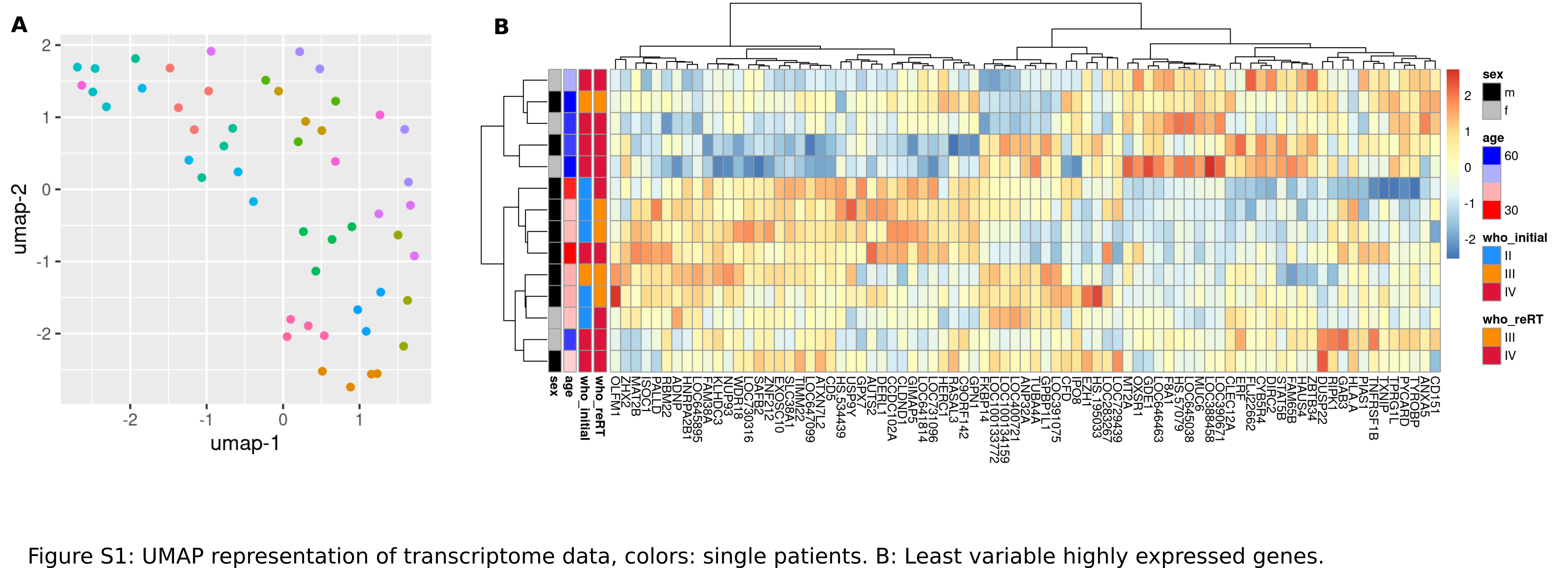

Supplement: Supplementary file 1 [file cancers-14-00684-s001.zip › cancers-1439087-supplementary/Figure S1.png]

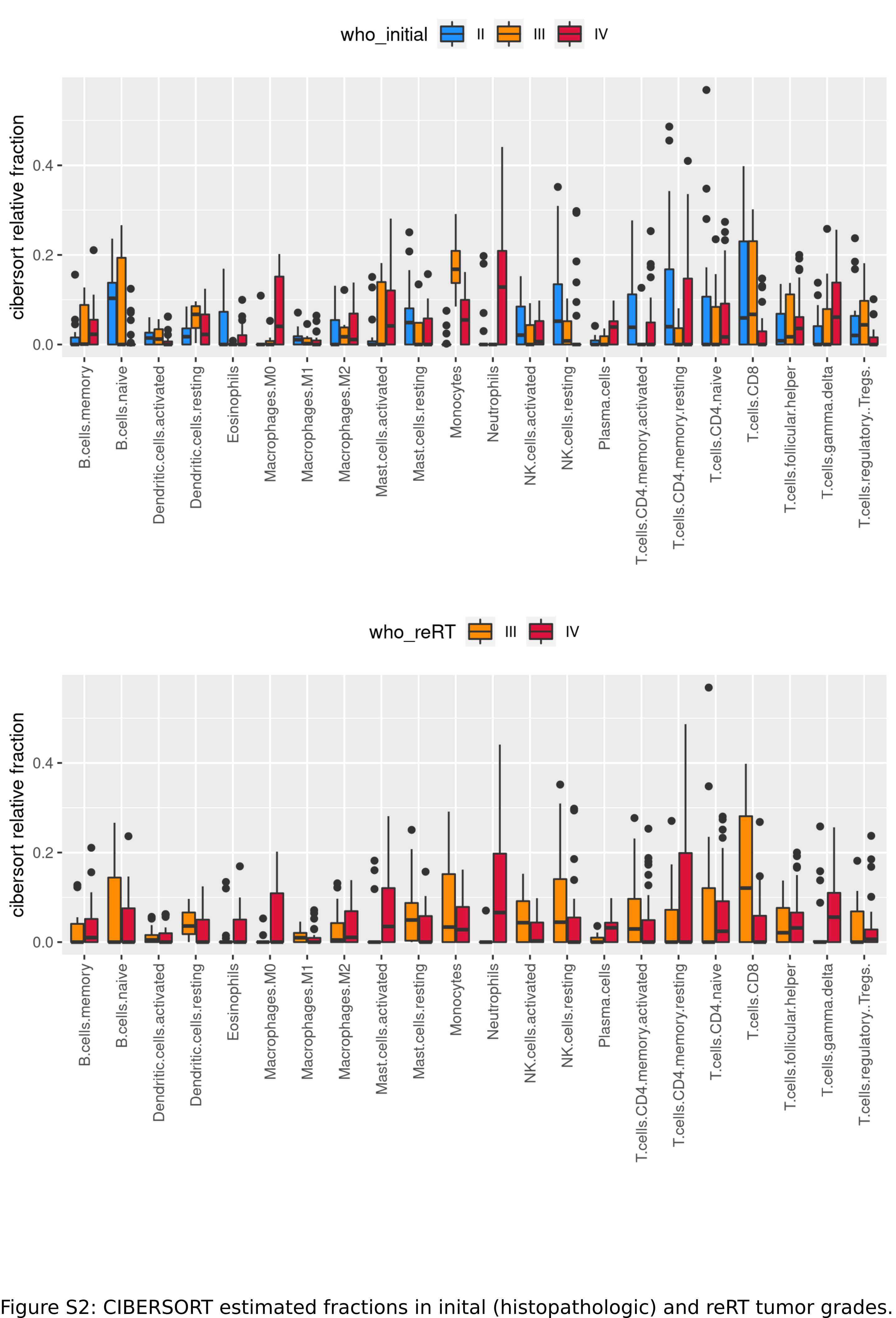

Supplement: Supplementary file 1 [file cancers-14-00684-s001.zip › cancers-1439087-supplementary/Figure S2.png]

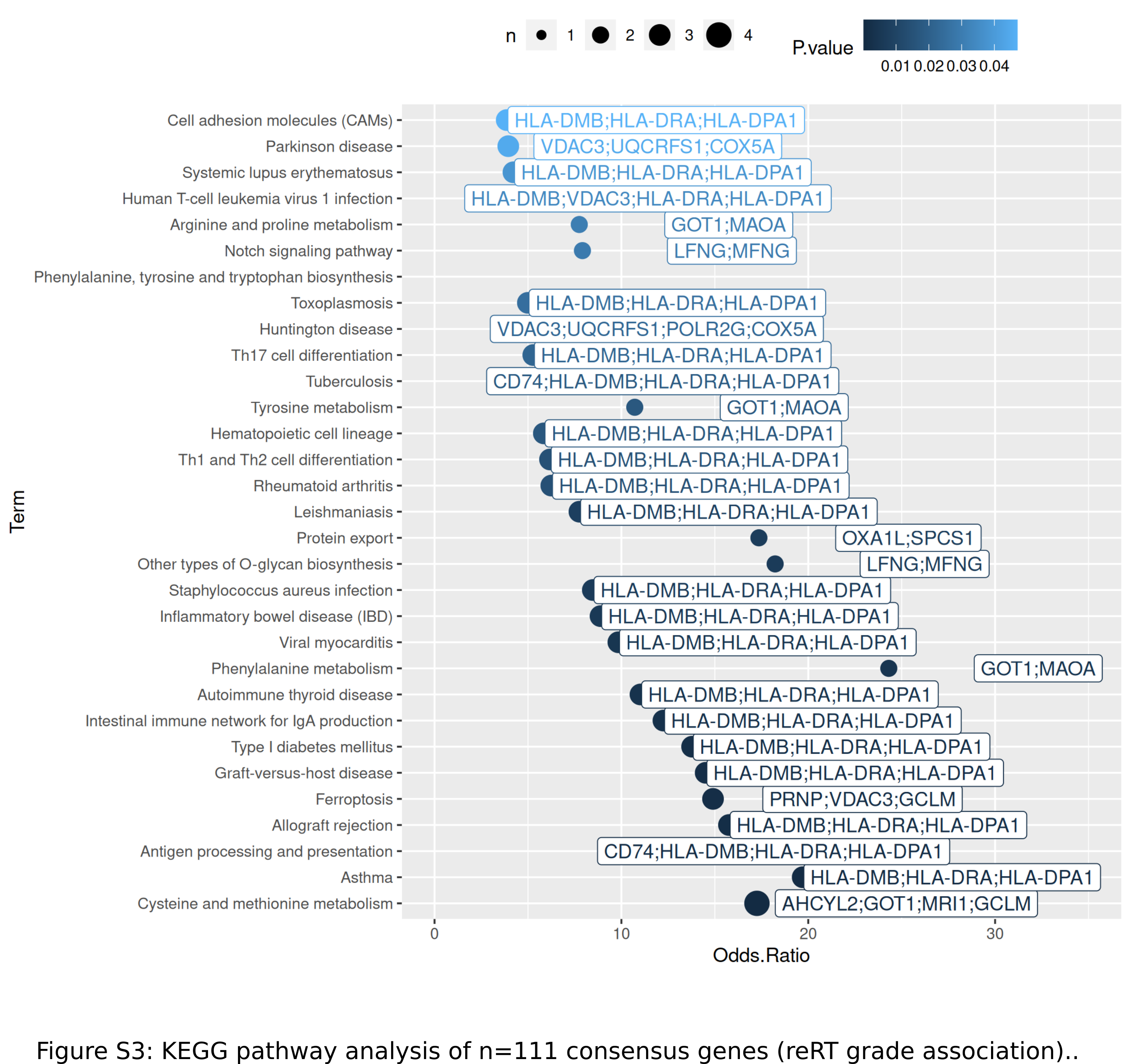

Supplement: Supplementary file 1 [file cancers-14-00684-s001.zip › cancers-1439087-supplementary/Figure S3.png]

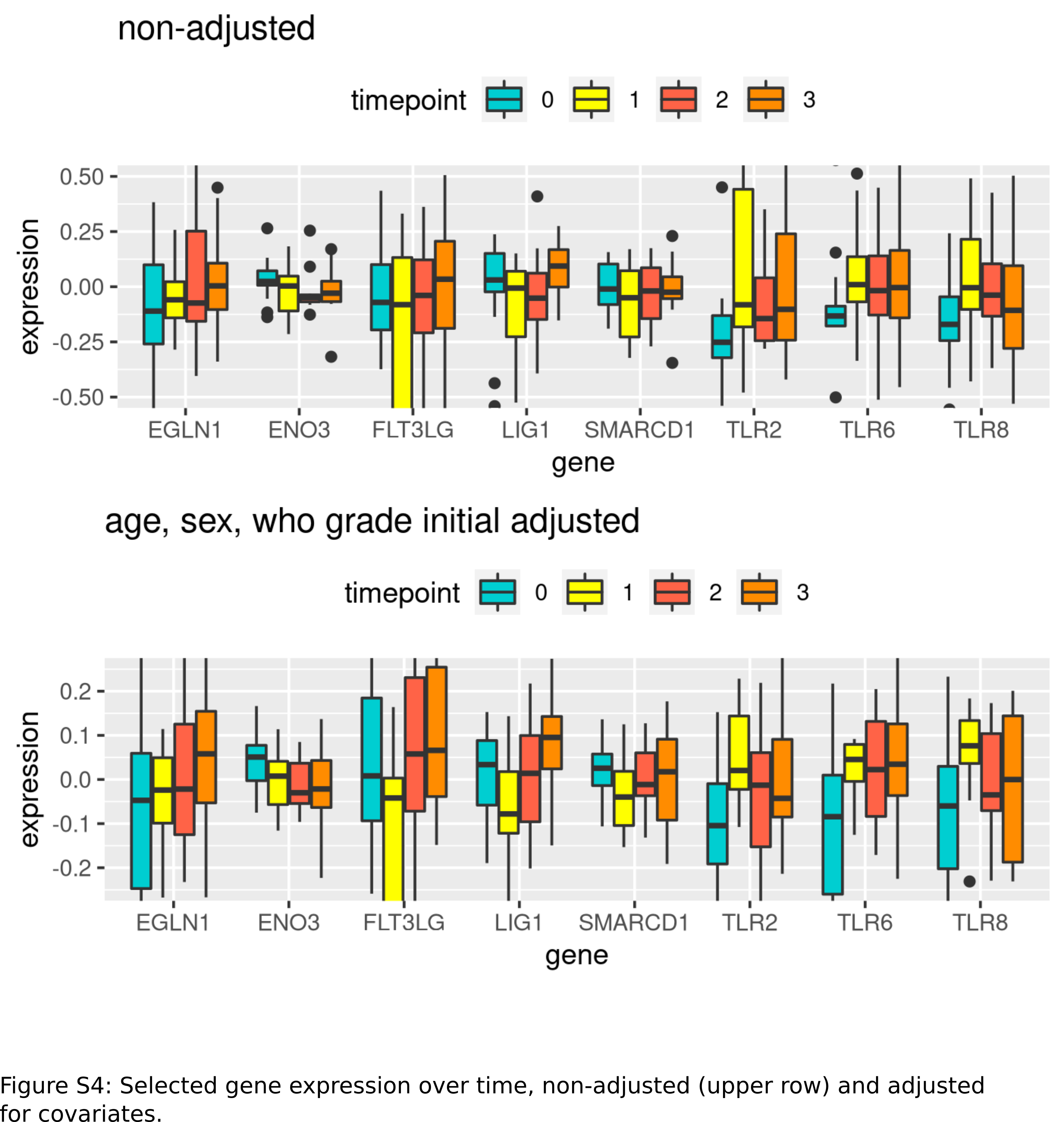

Supplement: Supplementary file 1 [file cancers-14-00684-s001.zip › cancers-1439087-supplementary/Figure S4.png]

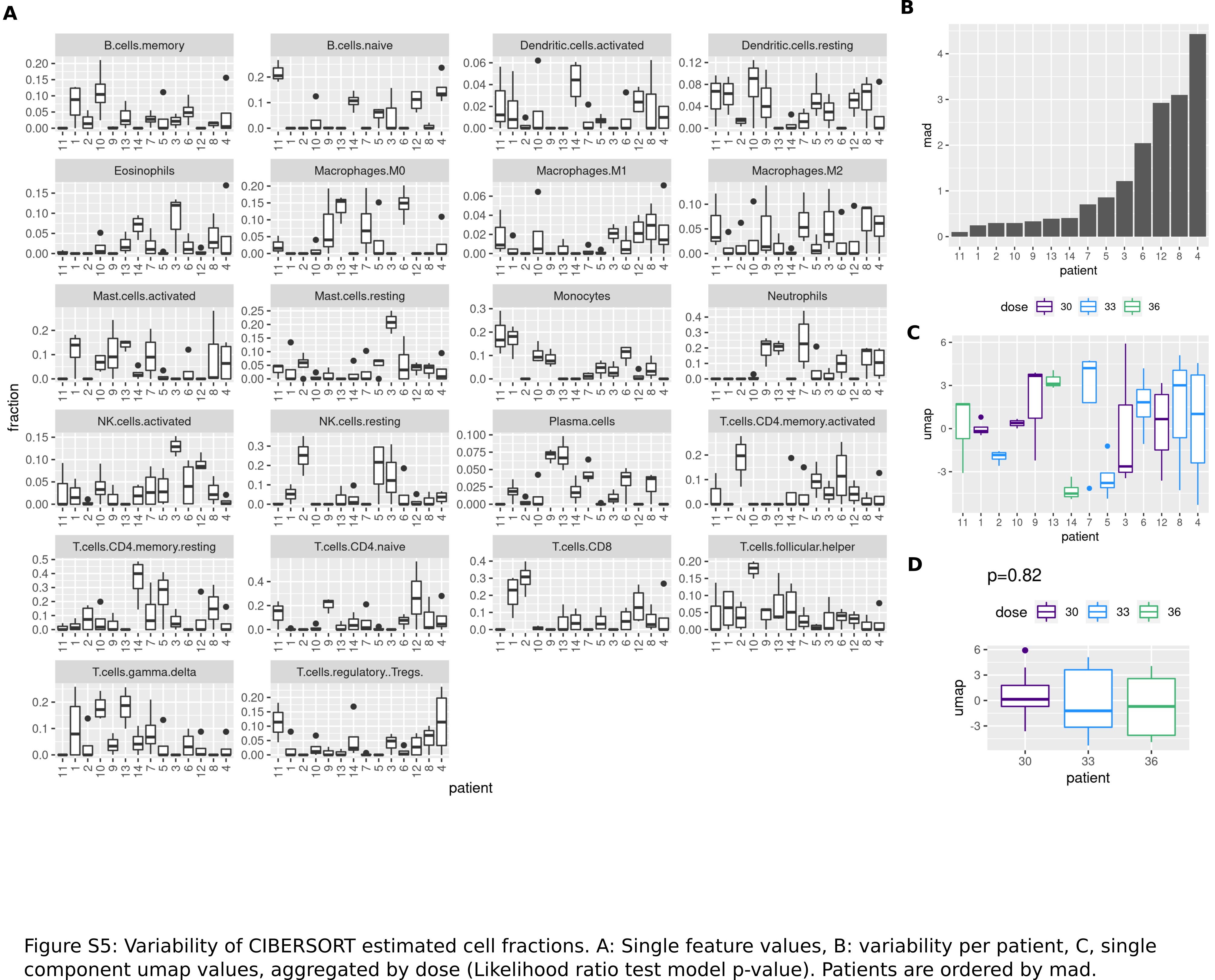

Supplement: Supplementary file 1 [file cancers-14-00684-s001.zip › cancers-1439087-supplementary/Figure S5.png]

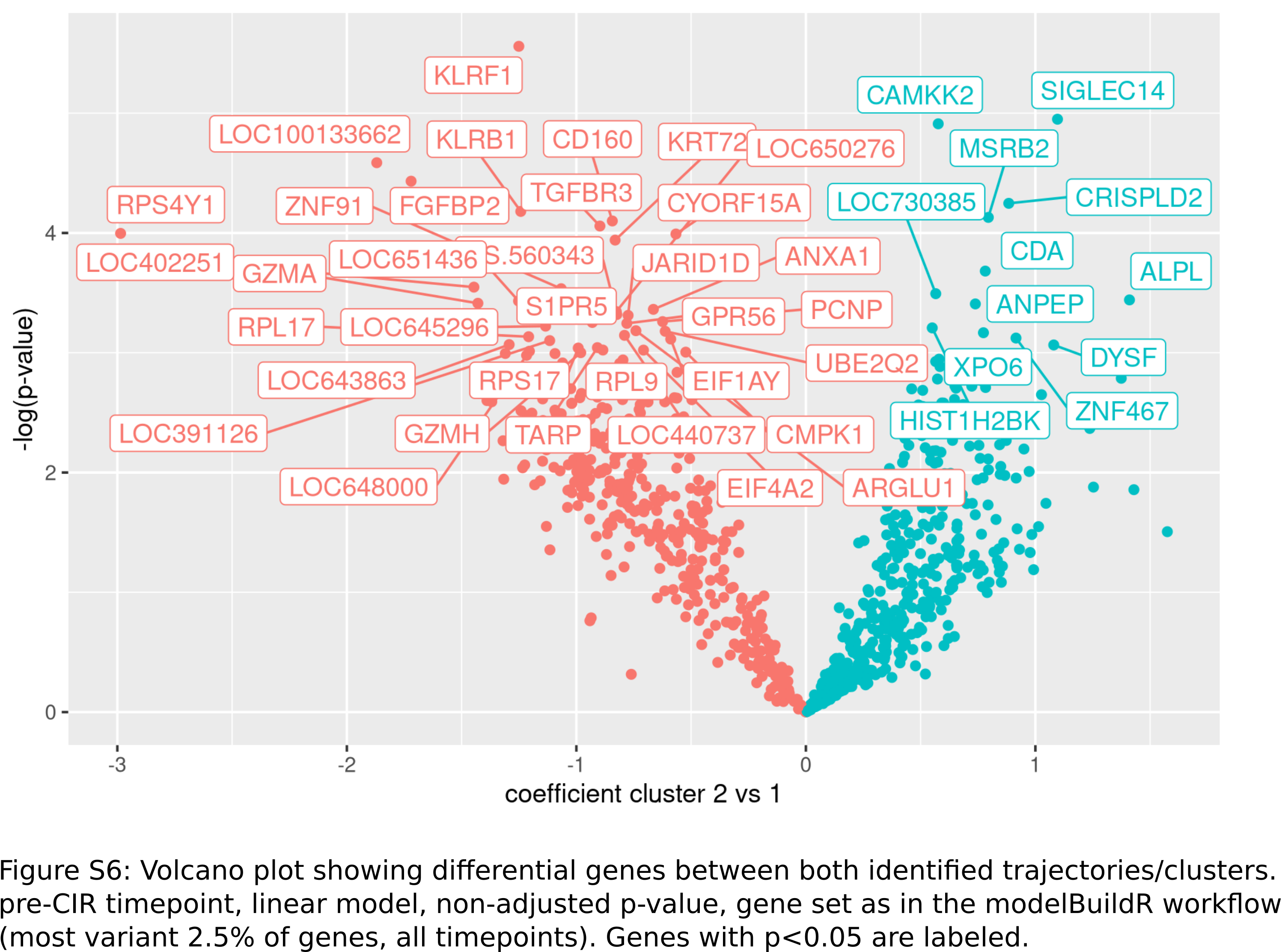

Supplement: Supplementary file 1 [file cancers-14-00684-s001.zip › cancers-1439087-supplementary/Figure S6.png]
